# Prognostic Implication of Liver Function Tests in Heart Failure With Preserved Ejection Fraction Without Chronic Hepatic Diseases: Insight From TOPCAT Trial

**DOI:** 10.3389/fcvm.2021.618816

**Published:** 2021-05-12

**Authors:** Weihao Liang, Xin He, Dexi Wu, Ruicong Xue, Bin Dong, Marvin Owusu-Agyeman, Jingjing Zhao, Linnuan Cai, Zhiyao You, Yugang Dong, Chen Liu

**Affiliations:** ^1^Department of Cardiology, The First Affiliated Hospital of Sun Yat-Sen University, Guangzhou, China; ^2^NHC Key Laboratory of Assisted Circulation, Sun Yat-sen University, Guangzhou, China; ^3^Zhongshan School of Medicine, Sun Yat-sen University, Guangzhou, China

**Keywords:** heart failure with preserved ejection fraction, liver function tests, prognosis, cholestasis, congestive hepatopathy

## Abstract

**Background:** Liver dysfunction is prevalent in patients with heart failure (HF), but the prognostic significance of liver function tests (LFTs) remains controversial. Heart failure with preserved ejection fraction (HFpEF) had been introduced for some time, but no previous study had focused on LFTs in HFpEF. Thus, we aim to evaluate the prognostic significance of LFTs in well-defined HFpEF patients.

**Methods and Results:** We conveyed a *post-hoc* analysis of the Treatment of Preserved Cardiac Function Heart Failure with an Aldosterone Antagonist Trial (TOPCAT). The primary outcome was the composite of cardiovascular mortality, HF hospitalization, and aborted cardiac arrest, and the secondary outcomes were cardiovascular mortality and HF hospitalization. In Cox proportional hazards models, aspartate transaminase (AST) and alanine transaminase (ALT) were not associated with any of the outcomes. On the contrary, increases in total bilirubin (TBIL) and alkaline phosphatase (ALP) were associated with increased risks of the primary outcome [TBIL: adjusted hazard ratio (HR), 1.17; 95% confidence interval (CI) 1.08–1.26; ALP: adjusted HR, 1.12; 95% CI 1.04–1.21], cardiovascular mortality (TBIL: adjusted HR, 1.16; 95% CI 1.02–1.31; ALP: adjusted HR, 1.16; 95% CI 1.05–1.28), and HF hospitalization (TBIL: adjusted HR, 1.22; 95% CI 1.12–1.33; ALP: adjusted HR, 1.12; 95% CI 1.03–1.23).

**Conclusion:** Elevated serum cholestasis markers TBIL and ALP were significantly associated with a poor outcome in HFpEF patients without chronic hepatic diseases, while elevated ALT and AST were not.

## Introduction

Liver dysfunction is prevalent in patients with chronic heart failure (CHF) (1). Both hypoperfusion due to reduced cardiac output and congestion secondary to volume and pressure overload could lead to hepatic injury (2). Although, it is known that CHF patients with severe hepatic dysfunction had a poor outcome (3), the prognostic value of abnormal liver function tests (LFTs) has not been established. Several studies focusing on this issue reported conflicting results. Some studies demonstrated strong prognostic values of increased serum aminotransferase (aspartate transaminase and alanine transaminase) in CHF patients (4, 5), while others found an association of worse clinical outcomes with the increase in cholestatic measurements, such as total bilirubin, alkaline phosphatase, and γ-glutamyltransferase, instead of aminotransferase (1, 6, 7).

It has been reported that patterns of abnormal LFTs were associated with congestion and hypoperfusion of the liver in the setting of heart failure CHF (8), suggesting that changes in LFTs might be indicators of hemodynamic disturbance in CHF. Recently, HF with preserved ejection fraction (HFpEF) has been recognized to be a distinct disease entity from HF with reduced ejection fraction (HFrEF) (9); however, no previous study had focused on LFTs in HFpEF patients.

Therefore, this study aimed at evaluating the prognostic implication of LFTs, including aspartate transaminase (AST), alanine transaminase (ALT), total bilirubin (TBIL), and alkaline phosphatase (ALP), in well-defined HFpEF patients. To avoid the influence of hepatic dysfunction, we further excluded patients with known hepatic diseases in the present study.

## Materials and Methods

### Study Population

This was a *post-hoc* analysis of data from the Treatment of Preserved Cardiac Function Heart Failure with an Aldosterone Antagonist Trial (TOPCAT), which was a phase 3, multicenter, international, randomized, double-blinded, placebo-controlled trial. Totally, 3,445 HFpEF patients were included and randomized to receive spironolactone or placebo treatment. Specifically, patients with known chronic hepatic diseases with AST or ALT >3.0 times the upper limit of normal were excluded from the study. The design and results of TOPCAT were published elsewhere (10, 11). Patients or the public was not involved in the design, or conduct, or reporting, or dissemination of our research.

Data analyzed in this study were obtained from the National Institutes of Heart, Lung, and Blood Institute's Biologic Specimen and Data Repository Information Coordinating Center. Data from Russia and Georgia were excluded because of concerns about the representativeness of HFpEF patients in these two countries (12), leaving 1,767 patients from the Americas for analysis. Among these patients, those with missing data on LFTs or any of the potential confounders mentioned below were excluded. No exclusion criteria for drugs that might affect liver function was applied. Finally, there were 1,657 patients included in the analyses. The present study was approved by the Medical Ethics Commission of the First Affiliated Hospital of Sun Yat-sen University, China.

### Liver Function Tests

Serum AST, ALT, TBIL, and ALP were measured at baseline. Based on routine laboratory standards, the upper limits of normal were 35 U/L for AST and ALT, 1.0 mg/dl for TBIL, and 120 U/L for ALP (13).

### Outcome of Interest

The primary outcome was a composite of cardiovascular mortality, HF hospitalization, and aborted cardiac arrest. Secondary outcomes were cardiovascular mortality and HF hospitalization.

### Statistical Analysis

As patients with known chronic hepatic diseases were excluded from TOPCAT, most of the elevated LFT results did not exceed two times the upper limit of normal. Continuous variables were presented as mean ± SD and compared by Student's *T*-test. Categorical variables were presented as percentages and compared by chi-squared test. Kaplan–Meier curves with log-rank tests were performed to observe differences in primary and secondary outcomes between elevated vs. normal LFTs groups. Multivariate Cox proportional hazards models were used to evaluate the association of LFTs and clinical outcomes. To adjust for potential confounders, age, gender, race, New York Heart Association (NYHA) classification (III and IV vs. I and II), previous HF hospitalization, history of myocardial infarction, chronic obstructive pulmonary disease, diabetes mellitus, smoking, alcohol use, heart rate, systolic blood pressure, body mass index, ejection fraction, hemoglobin, estimated glomerular filtration rate, and randomized treatment were also included in the models as covariates. Five proportional hazards models were established to comprehensively evaluate the prognostic significance of each liver function measurement. In model 1, liver function measurement was included as a categorical variable (elevated vs. normal). In model 2, the measurements were included as continuous variables. Variables in models 3 and 4 were the same as models 1 and 2, but to rule out the influence of extreme values, patients with liver function measurement >2 times the upper limit of normal were excluded. To explore potential non-linear relation, in model 5, liver function measurements were included as continuous variables with restricted cubic remodeling. Three knots were located to the 10th, 50th, and 90th percentiles following Harrell's suggestion (14). Liver function measurements were also limited to two times the upper limit of normal because restricted cubic remodeling could be affected by extreme values. Baseline brain natriuretic peptide (BNP) or N-terminal-pro-BNP (NT-proBNP) levels were available in only 992 patients; thus, we calculated standardized *z*-scores of BNP and NT-proBNP as previously reported (15) and included them in multivariate models as a sensitivity analysis. Statistical analyses were performed using STATA (version 13) and IBM SPSS (version 25). Hazard ratios (HRs), confidence intervals (CIs), and *P*-values were reported. *P* < 0.05 was regarded as statistical significance.

## Results

### Baseline Characteristics

Among patients included in analyses, proportions of patients with elevated AST, ALT, TBIL, and ALP were 12.3, 16.8, 11.4, and 19.1%, respectively. Baseline characteristics are summarized in Table 1. Compared with the normal groups, patients with elevated AST and ALT were younger, had lower systolic blood pressure, and higher hemoglobin levels. Besides, patients with elevated ALT also had higher proportions of NYHA I or II and alcohol use. Patients with elevated TBIL were older, more likely to be male and non-diabetic, with a lower systolic blood pressure but a higher hemoglobin level. Those with elevated ALP were younger, more likely to be female with previous HF hospitalization, and had a faster heart rate. However, alcohol use, a history of chronic obstructive pulmonary disease, and use of statins were less common than the normal group.

**Table 1:** Baseline characteristics of patients with and without abnormal liver function tests.

	**AST**		**ALT**		**TBIL**		**ALP**	
	**Normal**	**Elevated**	**Normal**	**Elevated**	**Normal**	**Elevated**	**Normal**	**Elevated**
	***N* = 1,454**	***N* = 203**	***N* = 1,378**	***N* = 279**	***N* = 1,468**	***N* = 189**	***N* = 1,340**	***N* = 317**
Age, y	71.9 ± 9.6	70.1 ± 10.2^*^	72.1 ± 9.7	69.4 ± 9.5^*^	71.5 ± 9.7	73.1 ± 9.9^*^	71.9 ± 9.7	70.6 ± 9.8^*^
Male, *n* (%)	721 (49.6)	106 (52.2)	674 (48.9)	153 (54.8)	702 (47.8)	125 (66.1)^*^	687 (51.3)	140 (44.2)^*^
Caucasian, *n* (%)	1,144 (78.7)	159 (78.3)	1,076 (78.1)	227 (81.4)	1,147 (78.1)	156 (82.5)	1,056 (78.8)	247 (77.9)
Previous HF hospitalization, *n* (%)	853 (58.7)	118 (58.1)	807 (58.6)	164 (58.8)	868 (59.1)	103 (54.5)	768 (57.3)	203 (64.0)^*^
NYHA III and IV, *n* (%)	501 (34.5)	73 (36.0)	493 (35.8)	81 (29.0)^*^	505 (34.4)	69 (36.5)	456 (34.0)	118 (37.2)
EF, %	58.3 ± 7.7	57.9 ± 7.8	58.2 ± 7.7	58.4 ± 7.8	58.3 ± 7.8	57.9 ± 7.4	58.1 ± 7.7	58.8 ± 8.1
Spironolactone arm, *n* (%)	734 (50.5)	95 (46.8)	696 (50.5)	133 (47.7)	741 (50.5)	88 (46.6)	660 (49.3)	169 (53.3)
Myocardial infraction, *n* (%)	301 (20.7)	37 (18.2)	292 (21.2)	46 (16.5)	302 (20.6)	36 (19.0)	278 (20.7)	60 (18.9)
Diabetes, *n* (%)	658 (45.3)	77 (37.9)	617 (44.8)	118 (42.3)	677 (46.1)	58 (30.7)^*^	595 (44.4)	140 (44.2)
COPD, *n* (%)	243 (16.7)	32 (15.8)	227 (16.5)	48 (17.2)	245 (16.7)	30 (15.9)	243 (18.1)	32 (10.1)^*^
Current smoking, *n* (%)	88 (6.1)	16 (7.9)	87 (6.3)	17 (6.1)	93 (6.3)	11 (5.8)	84 (6.3)	20 (6.3)
Alcohol use, *n* (%)	372 (25.6)	64 (31.5)	344 (25.0)	92 (33.0)^*^	380 (25.9)	56 (29.6)	368 (27.5)	68 (21.5)^*^
Heart rate, bpm	69.0 ± 11.0	69.2 ± 12.5	69.0 ± 11.1	69.0 ± 11.4	68.9 ± 11.1	70.2 ± 11.4	68.3 ± 11.0	71.9 ± 11.4^*^
SBP, mmHg	128.2 ± 15.8	122.7 ± 15.8^*^	127.9 ± 15.8	125.8 ± 16.2^*^	127.9 ± 15.9	124.9 ± 15.5^*^	127.3 ± 16.0	128.6 ± 15.3
BMI, kg/m^2^	33.9 ± 8.0	32.8 ± 8.6	33.6 ± 8.0	34.2 ± 8.4	33.9 ± 8.1	32.7 ± 8.2	33.7 ± 8.1	33.7 ± 8.1
Hemoglobin, g/dL	12.8 ± 1.6	13.1 ± 1.7^*^	12.7 ± 1.6	13.5 ± 1.6^*^	12.8 ± 1.6	13.2 ± 2.0^*^	12.9 ± 1.6	12.9 ± 1.7
eGFR, ml/min	64.2 ± 21.4	66.9 ± 22.8	64.2 ± 21.8	66.3 ± 20.3	64.8 ± 21.9	62.3 ± 18.9	64.4 ± 20.7	64.9 ± 25.1
Use of statins	946 (65.1)	128 (63.1)	886 (64.3)	188 (67.4)	957 (65.2)	117 (61.9)	894 (66.7)	180 (56.8)^*^

*HF, heart failure; NYHA, New York Heart Association Classification; EF, ejection fraction; COPD, chronic obstructive pulmonary disease; SBP, systolic blood pressure; BMI, body mass index; eGFR, estimated glomerular filtration rate; AST, aspartate aminotransferase; ALT, alanine aminotransferase; TBIL, total bilirubin; ALP, alkaline phosphatase*.

* *P < 0.05 when compared with the normal group*.

### Liver Function Tests and Clinical Outcomes

**Figure 1:**
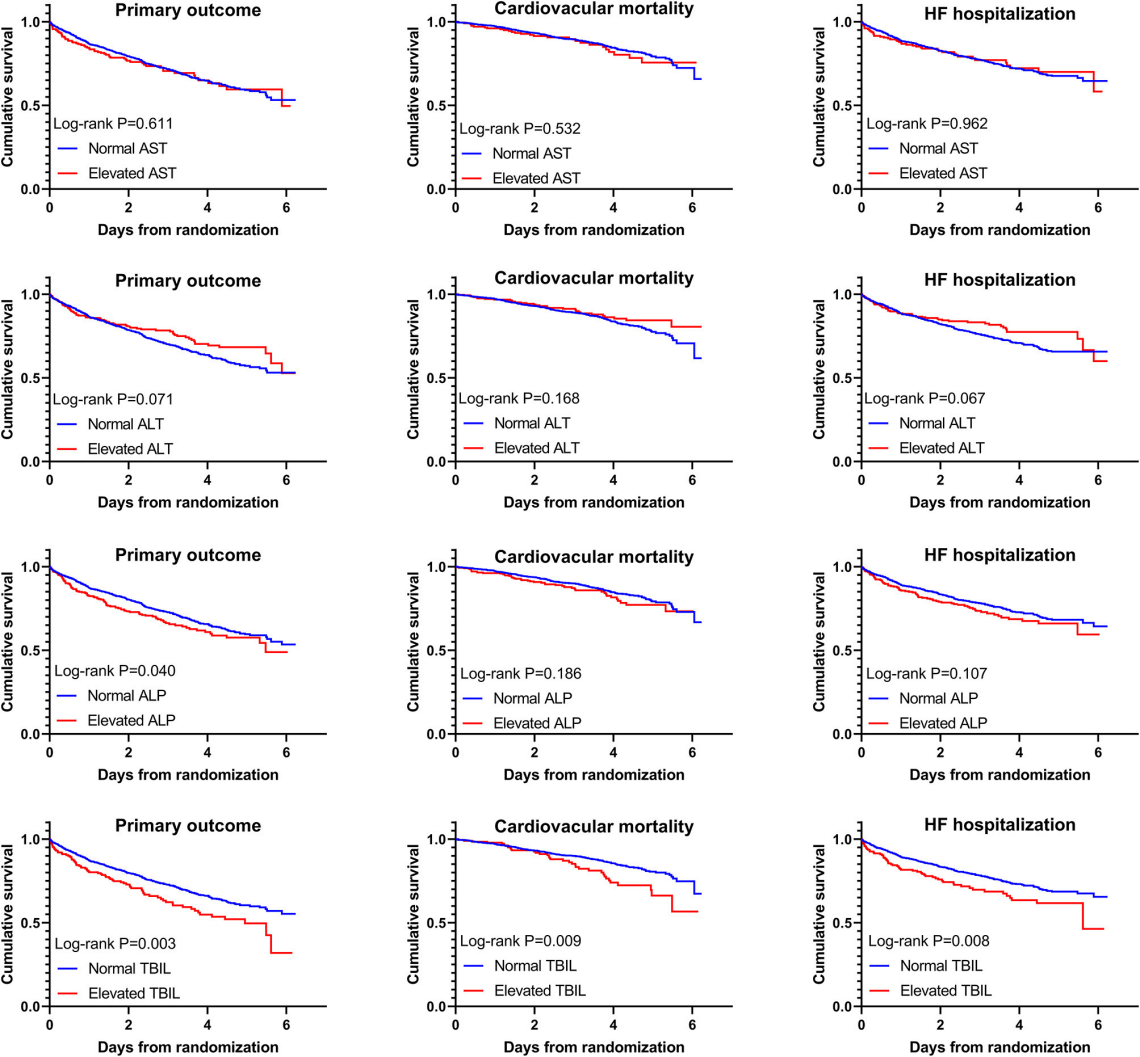
Kaplan–Meier curves with log-rank tests for comparison of elevated vs. normal aspartate transaminase (AST), alanine transaminase (ALT), total bilirubin (TBIL), and alkaline phosphatase (ALP) to primary and secondary outcomes.

Crude rates of outcome events are shown in Table 2. Kaplan–Meier curves (Figure 1) illustrated that elevated AST or ALT had a comparable risk of the primary outcome, cardiovascular mortality, and HF hospitalization compared with the normal groups. Elevated ALP had a higher risk of the primary outcome but comparable risks of cardiovascular mortality and HF hospitalization compared with the normal group, while patients with elevated TBIL had higher risks of the primary outcome, cardiovascular mortality, as well as HF hospitalization.

**Table 2:** Numbers and percentages of outcome events.

	**Normal**	**Elevated**	***P***
**The primary outcome**			
AST, *n/N* (%)	429/1,454 (29.5)	60/203 (29.6)	0.988
ALT, *n/N* (%)	417/1,378 (30.3)	72/279 (25.8)	0.137
TBIL, *n/N* (%)	415/1,468 (28.3)	74/189 (39.2)	0.002
ALP, *n/N* (%)	382/1,340 (28.5)	107/317 (33.8)	0.066
**Cardiovascular mortality**			
AST, *n/N* (%)	184/1,454 (12.7)	27/203 (13.3)	0.796
ALT, *n/N* (%)	180/1,378 (13.1)	31/279 (11.1)	0.373
TBIL, *n/N* (%)	175/1,468 (11.9)	36/189 (19.0)	0.006
ALP, *n/N* (%)	165/1,340 (12.3)	46/317 (14.5)	0.291
**HF hospitalization**			
AST, *n/N* (%)	331/1,454 (22.8)	44/203 (21.7)	0.728
ALT, *n/N* (%)	322/1,378 (23.4)	53/279 (19.0)	0.112
TBIL, *n/N* (%)	318/1,468 (21.7)	57/189 (30.2)	0.009
ALP, *n/N* (%)	294/1,340 (21.9)	81/317 (25.6)	0.167

*AST, aspartate aminotransferase; ALT, alanine aminotransferase; TBIL, total bilirubin; ALP, alkaline phosphatase*.

**Figure 2:**
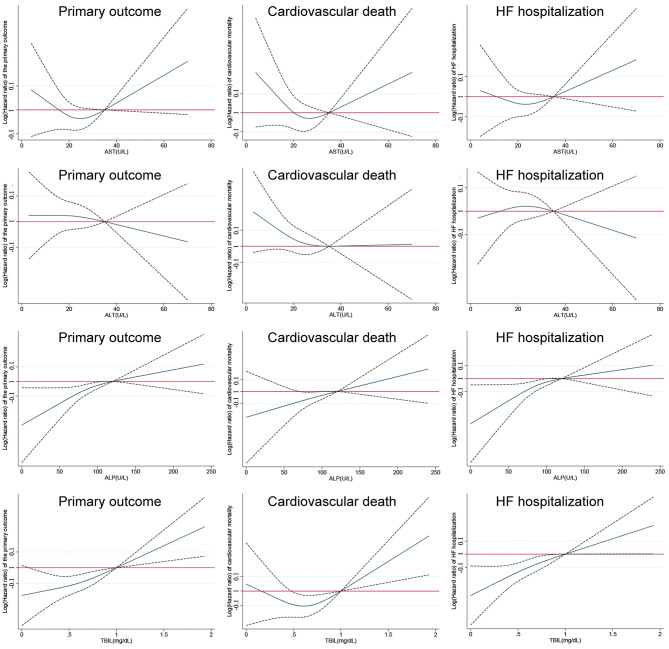
Restricted cubic remodeling of Cox proportional hazards models of aspartate transaminase (AST), alanine transaminase (ALT), total bilirubin (TBIL), and alkaline phosphatase (ALP) to primary and secondary outcomes (solid lines represent relative hazard ratios; dot lines represent upper and lower limits of 95% confidence intervals).

The results of model 1 are shown in Table 3, and those of model 2 are summarized in Table 4. In model 1, ALT and AST were not associated with any of the outcomes as categorical variables, while elevated TBIL was associated with increased risks of the primary outcome (HR, 1.51; 95% CI 1.17–1.94; *P* = 0.002), cardiovascular mortality (HR, 1.45; 95% CI 1.01–2.10; *P* = 0.047), and HF hospitalization (HR, 1.58; 95% CI 1.18–2.10; *P* = 0.002). Elevated ALP was associated with increased risk of the primary outcome (HR, 1.25; 95% CI 1.00–1.56; *P* = 0.046) but not cardiovascular mortality or HF hospitalization. When these markers were included as continuous variables in model 2, ALT and AST were still not associated with any of the outcomes, but increase in ALP and TBIL were associated with increased risks of the primary outcome (TBIL: HR, 1.17; 95% CI 1.08–1.26; *P* < 0.001; ALP: HR, 1.12; 95% CI, 1.04–1.21; *P* = 0.003), cardiovascular mortality (TBIL: HR, 1.16; 95% CI 1.02–1.31; *P* = 0.022; ALP: HR, 1.16; 95% CI, 1.05–1.28; *P* = 0.004), and HF hospitalization (TBIL: HR, 1.22; 95% CI, 1.12–1.33; *P* < 0.001; ALP: HR, 1.12; 95% CI, 1.03–1.23; *P* = 0.012). Models 3 and 4 excluded patients with LFTs >2 times the upper limit of normal, which yielded similar results except that associations of cardiovascular mortality and TBIL and ALP were no longer significant (Tables 3, 5). Although ALT and AST did not have significant results in the above Cox proportional hazards models, a non-linear association could not be excluded. Thus, we conducted the restricted cubic remodeling analysis; however, the result did not indicate non-linear relations of all four LFTs and outcomes as well (Figure 2), further, confirming that ALT and AST were not associated with the risks of outcomes. Interestingly, according to Figure 2, the positive association of ALP, TBIL, and outcome risk was not limited to abnormal results. Instead, this positive association began below the upper limit of the normal range.

**Table 3:** Associations of liver function tests as binary variable (Normal vs. Elevated) and clinical outcomes.

	**The primary outcome**		**Cardiovascular mortality**		**HF hospitalization**	
	Adjusted HR^*^ (95% CI)	***P***	Adjusted HR^*^ (95% CI)	***P***	Adjusted HR^*^ (95% CI)	***P***
**Model 1**						
Elevated AST	1.17 (0.89–1.54)	0.254	1.19 (0.79–1.80)	0.399	1.13 (0.82–1.56)	0.448
Elevated ALT	0.92 (0.71–1.18)	0.501	0.85 (0.58–1.26)	0.424	0.91 (0.68–1.23)	0.543
Elevated TBIL	1.51 (1.17–1.94)	0.002	1.45 (1.01–2.10)	0.047	1.58 (1.18–2.10)	0.002
Elevated ALP	1.25 (1.00–1.56)	0.046	1.28 (0.92–1.80)	0.147	1.24 (0.96–1.60)	0.096
**Model 3**						
Elevated AST	1.13 (0.83–1.53)	0.440	1.22 (0.77–1.92)	0.404	1.09 (0.77–1.55)	0.619
Elevated ALT	0.94 (0.72–1.23)	0.656	0.89 (0.59–1.33)	0.567	0.96 (0.70–1.30)	0.786
Elevated TBIL	1.41 (1.07–1.85)	0.015	1.40 (0.94–2.08)	0.100	1.40 (1.02–1.93)	0.038
Elevated ALP	1.15 (0.90–1.46)	0.259	1.14 (0.79–1.66)	0.483	1.14 (0.86–1.49)	0.360
**Sensitivity analysis (n** **=** **992)**						
Elevated AST	1.22 (0.84–1.77)	0.291	1.51 (0.86–2.67)	0.152	1.01 (0.64–1.58)	0.976
Elevated ALT	0.98 (0.70–1.37)	0.892	1.05 (0.63–1.75)	0.846	0.91 (0.61–1.35)	0.639
Elevated TBIL	1.33 (0.96–1.84)	0.085	1.34 (0.82–2.18)	0.241	1.37 (0.95–1.98)	0.094
Elevated ALP	1.35 (1.00–1.82)	0.054	1.47 (0.92–2.35)	0.108	1.28 (0.91–1.80)	0.160

* *Covariates for adjustment included age, gender, race, NYHA classification (III and IV vs. I and II), previous HF hospitalization, history of myocardial infarction, chronic obstructive pulmonary disease, diabetes mellitus, smoking, alcohol use, heart rate, systolic blood pressure, body mass index, ejection fraction, hemoglobin, estimated glomerular filtration rate, and randomized treatment*.

**Table 4:** Associations of liver function tests and clinical outcomes.

	**The primary outcome**		**Cardiovascular mortality**		**HF hospitalization**	
	Adjusted HR^*^ (95% CI)	***P***	Adjusted HR^*^ (95% CI)	***P***	Adjusted HR^*^ (95% CI)	***P***
AST	1.05 (0.96–1.15)	0.322	0.97 (0.84–1.12)	0.662	1.06 (0.96–1.18)	0.246
ALT	0.95 (0.86–1.04)	0.248	0.88 (0.75–1.02)	0.095	0.94 (0.84–1.05)	0.288
TBIL	1.17 (1.08–1.26)	<0.001	1.16 (1.02–1.31)	0.022	1.22 (1.12–1.33)	<0.001
ALP	1.12 (1.04–1.21)	0.003	1.16 (1.05–1.28)	0.004	1.12 (1.03–1.23)	0.012

*HR, hazard ratio; CI, confidence interval; HF, heart failure; AST, aspartate aminotransferase; ALT, alanine aminotransferase; TBIL, total bilirubin; ALP, alkaline phosphatase*.

* *Covariates for adjustment included age, gender, race, NYHA classification (III and IV vs. I and II), previous HF hospitalization, history of myocardial infarction, chronic obstructive pulmonary disease, diabetes mellitus, smoking, alcohol use, heart rate, systolic blood pressure, body mass index, ejection fraction, hemoglobin, estimated glomerular filtration rate, and randomized treatment. HRs were calculated as per standard deviation increase*.

**Table 5:** Associations of liver function tests and clinical outcomes after excluding patients with liver function test > 2 times upper limit of normal.

	**The primary outcome**		**Cardiovascular mortality**		**HF hospitalization**	
	Adjusted HR^*^ (95% CI)	***P***	Adjusted HR^*^ (95% CI)	***P***	Adjusted HR^*^ (95% CI)	***P***
AST	1.04 (0.93–1.17)	0.465	0.98 (0.82–1.17)	0.803	1.07 (0.94–1.22)	0.300
ALT	0.96 (0.85–1.07)	0.421	0.89 (0.75–1.06)	0.193	0.98 (0.86–1.11)	0.721
TBIL	1.24 (1.09–1.41)	0.002	1.19 (0.97–1.45)	0.088	1.31 (1.13–1.52)	<0.001
ALP	1.15 (1.03–1.30)	0.018	1.19 (1.00–1.42)	0.051	1.16 (1.02–1.33)	0.029

*HR, hazard ratio; CI, confidence interval; HF, heart failure; AST, aspartate aminotransferase; ALT, alanine aminotransferase; TBIL, total bilirubin; ALP, alkaline phosphatase*.

* *Covariates for adjustment included age, gender, race, NYHA classification (III and IV vs. I and II), previous HF hospitalization, history of myocardial infarction, chronic obstructive pulmonary disease, diabetes mellitus, smoking, alcohol use, heart rate, systolic blood pressure, body mass index, ejection fraction, hemoglobin, estimated glomerular filtration rate, and randomized treatment. HRs were calculated as per standard deviation increase*.

### Sensitivity Analysis

In the sensitivity analysis, we further adjusted BNP/NT-proBNP *z*-scores in multivariate analysis in 992 patients with available baseline BNP or NT-proBNP levels. When included as a categorical variable, none of the four LFTs was associated with the risk of the primary outcome, cardiovascular mortality, or HF hospitalization (Table 3). However, when included as a continuous variable, the results showed that the increase in TBIL was significantly associated with higher risks of the primary outcome (HR, 1.13; 95% CI 1.01–1.26; *P* = 0.031), cardiovascular mortality (HR, 1.20; 95% CI 1.02–1.42; *P* = 0.029), and HF hospitalization (HR, 1.17; 95% CI 1.05–1.32; *P* = 0.007). Similarly, the increase in ALP was significantly associated with higher risks of the primary outcome (HR, 1.19; 95% CI 1.05–1.35; *P* = 0.006), cardiovascular mortality (HR, 1.32; 95% CI 1.10–1.58; *P* = 0.002), and HF hospitalization (HR, 1.16; 95% CI 1.00–1.34; *P* = 0.044). AST and ALT were still not associated with any of the outcomes (Table 6).

**Table 6:** Associations of liver function tests and clinical outcomes in enrolled patients with BNP/NT-proBNP available (*n* = 992).

	**The primary outcome**		**Cardiovascular mortality**		**HF hospitalization**	
	Adjusted HR^*^ (95% CI)	***P***	Adjusted HR^*^ (95% CI)	***P***	Adjusted HR^*^ (95% CI)	***P***
AST	1.07 (0.95–1.21)	0.276	1.15 (0.96–1.38)	0.143	1.04 (0.90–1.20)	0.603
ALT	0.98 (0.86–1.11)	0.713	0.90 (0.72–1.12)	0.339	0.99 (0.86–1.15)	0.910
TBIL	1.13 (1.01–1.26)	0.031	1.20 (1.02–1.42)	0.029	1.17 (1.05–1.32)	0.007
ALP	1.19 (1.05–1.35)	0.006	1.32 (1.10–1.58)	0.002	1.16 (1.00–1.34)	0.044

*HR, hazard ratio; CI, confidence interval; HF, heart failure; AST, aspartate aminotransferase; ALT, alanine aminotransferase; TBIL, total bilirubin; ALP, alkaline phosphatase.*

* *Covariates for adjustment included age, gender, race, NYHA classification (III and IV vs. I and II), previous HF hospitalization, history of myocardial infarction, chronic obstructive pulmonary disease, diabetes mellitus, smoking, alcohol use, heart rate, systolic blood pressure, body mass index, ejection fraction, hemoglobin, estimated glomerular filtration rate, randomized treatment, and BNP/NT–proBNP z-scores. HRs were calculated as per standard deviation increase*.

## Discussion

In the present study, we demonstrated the independent prognostic significance of cholestatic LFTs—TBIL and ALP—instead of AST and ALT within a cohort of well-defined HFpEF patients without known hepatic diseases.

Abnormalities of LFTs were frequently seen in both chronic and acute HF (AHF) patients and closely related to hepatic perfusion and congestion (8). A recent review divided the abnormality of LFTs in HF into two subtypes according to different primary pathophysiology (16). Passive venous congestion that resulted in “congestive hepatopathy (CH),” which was supposed to be associated with increased bilirubin levels and high ALP levels from an increased central venous pressure (CVP) (17), was a common sign of congestive heart failure (18). Low cardiac output and arterial hypoperfusion resulted in “acute cardiogenic liver injury (ACLI),” which was associated with increased levels of AST and ALT in heart failure that was attributed to hepatocellular damage from decreased perfusion (17). As the liver's complex dual blood supply makes it relatively resistant to hepatocellular damage from hemodynamic perturbations, ACLI was expected only in cases of marked hypotension or hypoperfusion (16). Low cardiac output and arterial hypoperfusion were more common in HFrEF patients and/or AHF patients, which could lead to the elevation of ALT and/or AST (19–21). But in the TOPCAT trial, participants were chronic HFpEF patients, suggesting that they were unlikely to suffer from low cardiac output or arterial hypoperfusion. In terms of CH, elevated CVP could be transmitted directly to the hepatic veins, leading to hepatic congestion and impairment of the biliary system (22). Recently, Cogger et al. showed that hepatic congestion increased pressure within the hepatic sinusoid, leading to disruption of the liver sinusoidal endothelial cells and subsequent pressure increase in zonula occludens, which were the tight junctions between hepatocytes that separate the extravascular space from the bile canaliculus. Thus, disruption of the zonula occludens would expose the bile canaliculus directly to the sinusoidal blood causing the elevation of cholestasis markers (23). Additionally, Allen et al. (6) found that total bilirubin was significantly higher in patients who had evidence of volume overload on physical examination. CHF patients, unlike patients with AHF, did not frequently suffer from hypotension (18); therefore, changes in AST and ALT might be caused by other conditions or severe congestion, which leads to hepatocellular damage in CHF. By contrast, moderate congestion and elevated CVP were common in CHF (18), which could lead to CH and be reflected by the increases in TBIL and ALP. Some previous studies about LFTs in HF patients presented the same hypothesis, which found that TBIL and ALP were more likely to be associated with outcomes in CHF patients (1). Thus, the prognostic value of TBIL and ALP might represent the association of increased CVP and poor outcome (24). However, further study is needed to validate the hypothesis.

As discussed above, the changes in LFTs were associated with the alteration of hemodynamics in HF. Several studies have pointed out that the hemodynamic changes in HFpEF were different from HFrEF (25–27). Previous studies about LFTs in CHF patients showed inconsistent results. The average LVEF of these studies ranged from 28 to 51% (1, 5–7), implying that there was a large difference in the proportions of HFpEF and HFrEF in these studies. Additionally, Vyskocilova et al. (28) found that ALT and AST pattern predominated in the left-sided forward AHF (more likely presented by reduced EF), while cholestatic profile occurred mainly in the bilateral and right-sided AHF. The heterogeneity of CHF patients resulting from pooling HFpEF and HFrEF could be a reason for these inconsistent results. A recent *post-hoc* analysis of the PARADIGM-HF trial found that ALT was associated with worse prognosis in chronic HFrEF patients, as well as TBIL, but not AST (29). Of note, as they included chronic HFrEF patients, some of them could be with bilateral HF. Our study only focused on the HFpEF patients who were less likely to have left-sided HF to eliminate the heterogeneity caused by HF categories, and thus, the results were more convincing. Another reason for the conflicting results of previous studies could be the influence of coexisted hepatic diseases. None of the studies mentioned above (1, 4–7) set any exclusion criterion about the hepatic diseases. Indeed, proportions of abnormal LFTs at baseline differ significantly among studies mentioned above (1, 4–7). As discussed above, the elevated TBIL and ALP might reflect hemodynamic changes in our study. However, it would be a different story if abnormal LFTs were caused by hepatic diseases. As hepatic diseases could cause much larger changes in LFTs than hemodynamics of heart failure, the prognostic value of LFTs would be very hard to interpret. The present study had excluded patients with known chronic hepatic diseases, and further, in models 3 and 4, patients with potential unknown hepatic diseases at admission had also been excluded (those whose liver function measurement >2 times the upper limit of normal). Thus, the results were not confounded by coexisted hepatic diseases and revealed that TBIL and ALP, instead of ALT and AST, had significant prognostic value. As far as we know, this is the first study to evaluate the prognostic value of LFTs in sole HFpEF patients without chronic hepatic diseases. Sensitivity analysis further confirmed the independent prognostic value of TBIL and ALP from BNP and NT-proBNP.

However, there are some limitations to our study. We had no data on hemodynamic parameters (e.g., CVP) of enrolled patients and could not further investigate the relationship between LFTs and hemodynamic parameters. In addition, it was reported that TBIL was strongly correlated with GGT and its prognostic value lost in a multivariable model including GGT (1), but we had no data on GGT, and thus, this potential confounder could not be adjusted. Besides, all patients enrolled in the TOPCAT trial are with chronic HFpEF, so we could not compare the prognostic value of LFTs with patients with AHF or HFrEF.

## Conclusions

Among HFpEF patients without chronic hepatic diseases, elevated TBIL and ALP, two serum cholestasis markers, were significantly associated with poor outcome. On the contrary, AST and ALT had no prognostic significance. The results suggested a potential role of TBIL and ALP measurement in HFpEF. More studies are needed to validate the correlation of TBIL, ALP, and hemodynamic parameters in HFpEF.

## Data Availability Statement

Publicly available datasets were analyzed in this study. This data can be found here: https://biolincc.nhlbi.nih.gov/studies/.

## Ethics Statement

The studies involving human participants were reviewed and approved by Medical Ethics Commission of First Affiliated Hospital of Sun Yat-sen University. The ethics committee waived the requirement of written informed consent for participation.

## Author Contributions

WL, XH, YD, and CL: conceptualization. WL, XH, and DW: methodology. WL: software. RX, BD, and MO-A: validation. WL and XH: formal analysis. DW: investigation and original draft preparation. LC: resources. ZY: data curation. MO-A, YD, and CL: manuscript review and editing. JZ, YD, and CL: supervision. DW, RX, BD, JZ, YD, and CL: funding acquisition. All authors have read and agreed to the published version of the manuscript.

## Conflict of Interest

The authors declare that the research was conducted in the absence of any commercial or financial relationships that could be construed as a potential conflict of interest.
